# C-MYC Aberrations as Prognostic Factors in Diffuse Large B-cell Lymphoma: A Meta-Analysis of Epidemiological Studies

**DOI:** 10.1371/journal.pone.0095020

**Published:** 2014-04-16

**Authors:** Kuangguo Zhou, Danmei Xu, Yang Cao, Jue Wang, Yunfan Yang, Mei Huang

**Affiliations:** Department of Hematology, Tongji Hospital, Tongji Medical College, Huazhong University of Science and Technology, Wuhan, Hubei, P. R. China; Hokkaido University, Japan

## Abstract

**Objectives:**

Various studies have investigated the prognostic value of C-MYC aberrations in diffuse large B-cell lymphoma (DLBCL). However, the role of C-MYC as an independent prognostic factor in clinical practice remains controversial. A systematic review and meta-analysis were performed to clarify the clinical significance of C-MYC aberrations in DLBCL patients.

**Methods:**

The pooled hazard ratios (HRs) for overall survival (OS) and event-free survival (EFS) were calculated as the main effect size estimates. The procedure was conducted according to the Cochrane handbook and PRISMA guidelines, including the use of a heterogeneity test, publication bias assessment, and meta-regression, as well as subgroup analyses.

**Results:**

Twenty-four eligible studies enrolling 4662 patients were included in this meta-analysis. According to the nature of C-MYC aberrations (gene, protein, and mRNA), studies were divided into several subgroups. For DLBCL patients with C-MYC gene abnormalities, the combined HR was 2.22 (95% confidence interval, 1.89 to 2.61) for OS and 2.29 (95% confidence interval, 1.81 to 2.90) for EFS, compared to patients without C-MYC gene abnormalities. For DLBCL patients with overexpression of C-MYC protein and C-MYC mRNA, pooled HRs for OS were 2.13 and 1.62, respectively. C-MYC aberrations appeared to play an independent role among other well-known prognostic factors in DLBCL. Addition of rituximab could not overcome the inferior prognosis conferred by C-MYC.

**Conclusion:**

The present systematic review and meta-analysis confirm the prognostic value of C-MYC aberrations. Screening of C-MYC should have definite prognostic meaning for DLBCL stratification, thus guaranteeing a more tailored therapy.

## Introduction

Non-Hodgkin lymphoma (NHL) is the fifth most frequent cancer worldwide, in which diffuse large B-cell lymphoma (DLBCL) ranks the most common histologic subtype. DLBCL comprises a heterogeneous group with varied clinical and molecular features and different prognoses, despite uniform treatment. Recognition of the biological heterogeneity of DLBCL is thus of clinical importance [1]. The International Prognostic Index (IPI) is currently the most well-established index for risk stratification of DLBCL patients [2]. However, this prognostic index has limitations in reflecting the biologic or genetic features of DLBCL. Even within the same IPI risk group, substantial variability in clinical outcome has been observed. A significant improvement in overall survival of DLBCL has been achieved since rituximab (R) was used in combination with cyclophosphamide, doxorubicin, vincristine, and prednisone (CHOP) as the first-line chemotherapy regimen [3]. However, the prognosis in high-risk DLBCL patients remains dismal. Identification and validation of novel prognostic biomarkers may contribute to better stratification of DLBCL and guide optimal treatment.

To date, several prognostic biomarkers of lymphoma have been investigated, in which C-MYC is one of the most prominent factors [4]. The nature of the C-MYC aberrations included gene translocation, gene amplification, and C-MYC mRNA or C-MYC protein overexpression. C-MYC gene translocation is a hallmark of Burkitt lymphoma and can be detected in 5–17% of DLBCL patients [5]. Despite the advances achieved in the assessing prognostic significance of C-MYC in DLBCL, recent studies have implied the prognostic value of C-MYC was complicated by other factors and is far from straightforward. For example, C-MYC gene translocation was frequently found in DLBCL with concurrent translocations of BCL2 and/or BCL6, referred to as “double-hit” or “triple-hit” lymphomas, and has a dismal prognosis [6]. Although some studies have shown that the presence of C-MYC aberrations was significantly associated with shorter survival in DLBCL [7], [8], [9], [10], [11], [12], [13], [14], [15], [16], [17], [18], [19], [20], [21], [22], [23], [24], other studies failed to show such an association between C-MYC and worse prognosis [25], [26], [27], [28], [29], [30]. Therefore, the role of C-MYC as independent prognostic factors needs to be further addressed in well-designed clinical trials. On the other hand, although numerous studies had been conducted to explore the prognostic values of C-MYC, no meta-analysis has assessed the predictive role of C-MYC aberrations in DLBCL. In the present study, we have conducted the first comprehensive systematic review and meta-analysis regarding the impact of C-MYC aberrations on DLBCL patients.

## Materials and Methods

### Selection criteria

A literature search was conducted in PUBMED, EMBASE, and COCHRANE databases. All the studies published before 31 January 2014 were included. Search criteria used synonyms of the following terms variably combined: C-MYC, prognosis and diffuse large B-cell lymphoma. The search was restricted to human studies with no language limitations.

According to the PRISMA guidelines [31], studies included in this meta-analysis should meet the following criteria: (i) C-MYC aberrations in adult patients with primary DLBCL had been examined by fluorescent in situ hybridization (FISH), immunohistochemistry (IHC) or other techniques; (ii) detailed survival information was available; and (iii) the median follow-up time exceeded one year. In the screening and eligibility stage, patients with evidence of an indolent lymphoma, human immunodeficiency virus infection, or primary central nervous system disease were excluded. Because compared to other DLBCL patients, patients with evidence of these factors mentioned above had some distinct features. Moreover, all studies were carefully evaluated to identify duplicate patient populations. Criteria used to determine duplicate populations included the study period, hospital, treatment information, and any additional inclusion items. However, subsequent reports containing new data on prognostic factors or survival were also incorporated into pooled analyses of the specific point.

### Quality assessment

Two investigators (KGZ and DMX) independently evaluated the methodological quality of the studies twice, applying the Newcastle-Ottawa Quality Assessment scale for case-control and cohort studies [32]. According to the quality scales, if a study met a requirement, then it gained a score of 1; otherwise, it gained a score of 0. REMARK guidelines, which provide a useful start for assessing tumor prognostic markers, were used to help identify study bias [33]. When discrepancies between investigators occurred, a third investigator (MH) conducted an additional evaluation.

### Data extraction

Baseline characteristics of the included studies, such as follow-up time, were independently recorded on a spreadsheet. In each study, both overall survival (OS) and event-free survival (EFS) were considered endpoints for survival analysis. We assessed the prognostic impact of C-MYC using hazard ratios (HRs) and their 95% confidence intervals (95%CIs) as the main effect size estimate. For each study, HR was estimated by a method depending on the data provided in the publication. For those studies that reported the value of HR and its standard error straightforward, these data would be extracted directly. For those studies that did not report the HR but provided sufficient data on survival, the log HRs and variances were estimated based on the methodology published previously [34]. An HR greater than 1 implied a worse survival for those patients with C-MYC. Otherwise, if the value of HR was smaller than 1, a better prognosis was indicated for the group with C-MYC. Additionally, if a 95% CI for the hazard ratio included the null value of 1, then this estimate of HR was not statistically significant. If the value of HR could be obtained from both multivariate and univariate analyses, we extracted the HR from multivariate analyses.

The secondary effect size estimate was odds ratio (OR), which reflects the possible association between the previously well-known prognostic factors with C-MYC status in DLBCL, and *P* < 0.05 was considered statistically significant.

### Statistical analysis

The procedure was conducted according to the Cochrane handbook, including a sensitivity test, heterogeneity test, publication bias test, and meta-regression, as well as subgroup analyses. Heterogeneity among studies was evaluated using the Cochran's Q test as well as the *I^2^* index. If the heterogeneity was substantial (*I^2^* ≥50%), the random effects model was performed. Otherwise, the fixed effects model was used. Meta-regression analysis of multiple covariates was also performed to examine the sources of heterogeneity, particularly the differences in the length of follow-up time [35], [36]. Begg's and Egger's test were used to reveal possible publication bias [37], [38]. A sensitivity analysis was performed to assess the stability. The inverse of the estimates' variance was used as study weight, such that larger studies tended to contribute more than smaller studies to the weighted average. The weighting factor was 1/(standard error)^2^ in our study. All calculations were performed using Stata 12.0 (StataCorp, College Station, TX).

## Results

### Characteristics of the included studies

**Figure 1:**
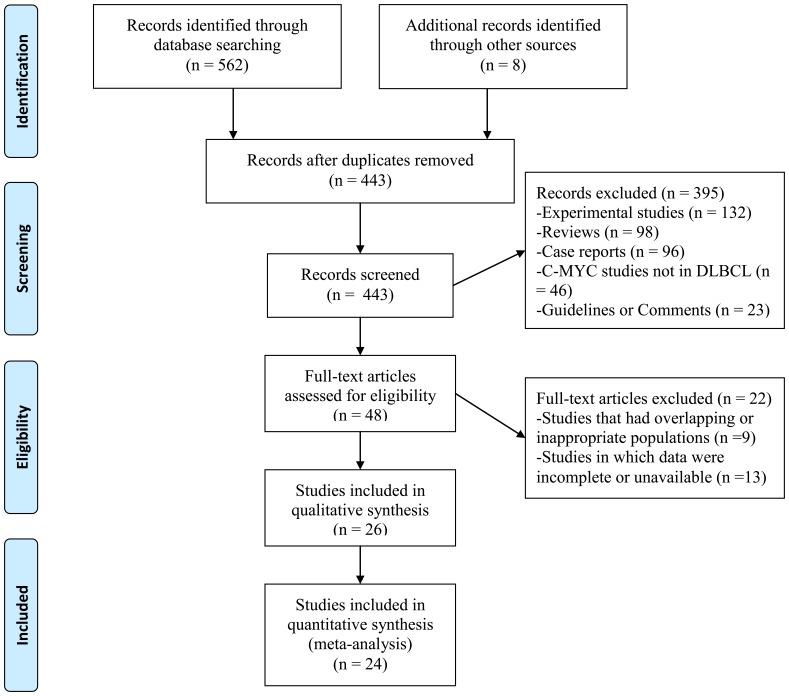
PRISMA flow chart of study selection.

The selection procedure of eligible studies was shown as a flow chart (Figure 1). Twenty-four articles finally met the inclusion criteria, and their data were summarized in Table 1. The sample size in each study varied from 24 to 562, with a median follow-up time ranging from 24 to 64 months. Eight studies had reported the effect of C-MYC on EFS endpoints, and twenty-two studies on OS endpoints. Among these publications, the nature of the C-MYC aberrations included gene translocation, gene amplification, and C-MYC mRNA or C-MYC protein overexpression. The HRs and 95%CIs were therefore calculated according to the manifestation of C-MYC aberrations (gene, protein, and mRNA). In particular, Gupta *et al.* and Horn *et al.* evaluated the association of survival endpoints with both C-MYC gene translocation and C-MYC protein overexpression in DLBCL [11], [26]. In Johnson *et al.* study, high levels of both C-MYC mRNA and C-MYC protein were both investigated [27]. Based on the nature of C-MYC aberrations, the prognostic values of C-MYC aberrations were analyzed in corresponding subgroups. The hazard ratios were carefully retrieved. Fifteen studies had the direct data for HR. For the other nine studies, the hazard ratios were extrapolated from the graphical survival distributions.

**Table 1:** Features summary of the eligible studies in the meta-analysis.

Study	Year	Region	Number of patients	Detection rate	Detection method	Main treatment	Outcome	Median follow-up months (minimum, maximum)	HR	Study quality
Klapper	2008	Germany	177	7.9%	FISH	CHOP-like	EFS, OS	29 (1, 71)	Reported in text	9
Yoon	2008	Korea	156	16.1%	FISH	Mainly CHOP-like	OS	42 (3, 76)	Reported in text	7
Niitsu	2009	Japan	252	11%	Conventional G-banding technique	CyclOBEAP or CHOP-like	EFS, OS	64 (18–90)	Reported in text	7
Savage	2009	Canada	135	8.8%	FISH	R-CHOP	EFS, OS	36 (0.8, 84)	Reported in text	8
Barrans	2010	Britain	245	14%	FISH	R-CHOP	OS	24 (1, 41)	Reported in text	7
Zhang	2011	China	106	12.3%	FISH	Mainly CHOP-like	OS	35 (4, 104)	Reported in text	6
Hummel	2006	Germany	146	16.6%	FISH	Mix treatment	OS	60 (0.1, 209)	Reported in text	8
Cuccuini	2012	France	161	17%	FISH	R-DHAP or R-ICE	EFS, OS	30 (2, 76)	Reported in text	8
Green	2012	Denmark	193	11%	FISH	R-CHOP	OS	47 (1, 102)	Reported in text	8
Kramer	1998	Netherlands	151	7%	Southern blot	CHOP-like	OS	40 (1, 96)	Data-extrapolated	7
Akasaka	2000	Japan	203	11.8%	LD-PCR	CHOP-like	OS	49 (29, 118)	Data-extrapolated	8
Akyurek	2012	Turkey	239	6%	FISH	R-CHOP	OS	26 (2, 96)	Data-extrapolated	7
Kawasaki	2001	Japan	137	10.2%	Southern blot	CHOP-like	OS	25 (0.1, 99)	Data-extrapolated	8
McClure	2005	America	76	5%	FISH	Mix treatment	OS	32 (1, 219)	Reported in text	7
Saez	2003	Spain	48	27%	RT-PCR	CHOP	EFS, OS	62 (12, 110)	Reported in text	7
Rimsza	2008	Mix	208	NA	qNPA	R-CHOP and CHOP analyzed separately	OS	26 (0.6, 70)	Reported in text	8
Kojima	2013	Japan	100	10%	FISH	R-CHOP	OS	49 (2, 118)	Reported in text	8
Horn	2013	Germany	442	31.8%/8.8%	IHC/FISH	Mix treatment	EFS, OS	29 (4, 64)	Reported in text	8
Johnson	2012	Mix	307	33%/NA	IHC/Microarray	R-CHOP	OS	49 (6, 136)	Reported in text	8
Aukema	2013	Germany	562	11.5%	FISH	Mix treatment	OS	63 (3, 120)	Data-extrapolated	8
Perry	2014	Mix	106	65%	IHC	Mix treatment	OS	54(7, 145)	Data-extrapolated	7
Tzankovet	2013	America	432	9%	FISH	R-CHOP	EFS	50(10, 95)	Data-extrapolated	8
Kluk	2012	America	56	17.8%	IHC	R-CHOP	OS	42 (2, 87)	Data-extrapolated	9
Gupta	2012	America	24	29%/10.4%	IHC/FISH	R-CHOP/epratuzumab	EFS	24 (3, 55)	Data-extrapolated	7

Abbreviations: HR, hazard ratio; OS, overall survival; EFS, event-free survival; IHC, immunohistochemistry; FISH, interphase fluorescent *in situ* hybridization; RT-PCR, reverse transcription-polymerase chain reaction; qNPA, quantitative nuclease protection assay; LD-PCR, long-distance polymerase chain reaction; CHOP, cyclophosphamide, doxorubicin, vincristine, and prednisone; R-CHOP, rituximab, cyclophosphamide, doxorubicin, vincristine, and prednisone; CyclOBEAP, cyclophosphamide, vincristine, bleomycin, etoposide, doxorubicin, and prednisone; R-DHAP, rituximab, dexamethasone, aracytine, and cisplatin; R-ICE, rituximab, ifosfamide, etoposide, and carboplatin; NA, not available.

### Meta-analysis for the associations between previously well-known prognostic factors with C-MYC aberrations

Six studies investigated the correlation of C-MYC with the cell-of-origin classified subgroups, including the germinal center B-cell-like (GCB) and non-GCB groups [39]. Meta-analysis revealed C-MYC were significantly more prevalent in the GCB type than in the non-GCB type (*P*  =  0.0002). The correlations of the previously well-known prognostic factors with C-MYC aberrations are summarized in Table 2. Patients with C-MYC presented more frequently with poor performance status (PS), an elevated level of lactate dehydrogenase (LDH), a high Ki-67 proliferation index and poor IPI. However, the frequencies of extranodal lesion, Ann Arbor stages III∼IV or bone marrow involvement were not significantly different between DLBCL patients with and without C-MYC aberrations (*P* > 0.05).

**Table 2:** Associations between the previously well-known prognostic factors with C-MYC aberrations.

	Studies	Pooled ORs	95% CI	*P*-value	*I^2^* value
Ann Arbor stage (III∼IV)	10	1.15	[0.82, 1.63]	0.4137	23.8%
Bone marrow involvement	4	1.36	[0.73, 2.52]	0.3349	45.9%
IPI (3∼5)	10	1.74	[1.21, 2.51]	0.0028	23.5%
Ki-67 index (> 80∼90%)	4	2.49	[1.13, 5.47]	0.0236	46.2%
LDH level (> Normal)	7	2.61	[1.63, 4.19]	0.0001	0%
Extranodal lesion (> 1)	9	1.07	[0.71, 1.63]	0.7300	36.8%
Performance status (score > 1)	7	2.17	[1.38, 3.41]	0.0008	0%

Abbreviations: IPI, international prognostic index; LDH, lactate dehydrogenase; OR, odd ratio; CI, confidence interval.

### Meta-analysis for the prognostic effects of C-MYC aberrations in DLBCL patients

**Figure 2:**
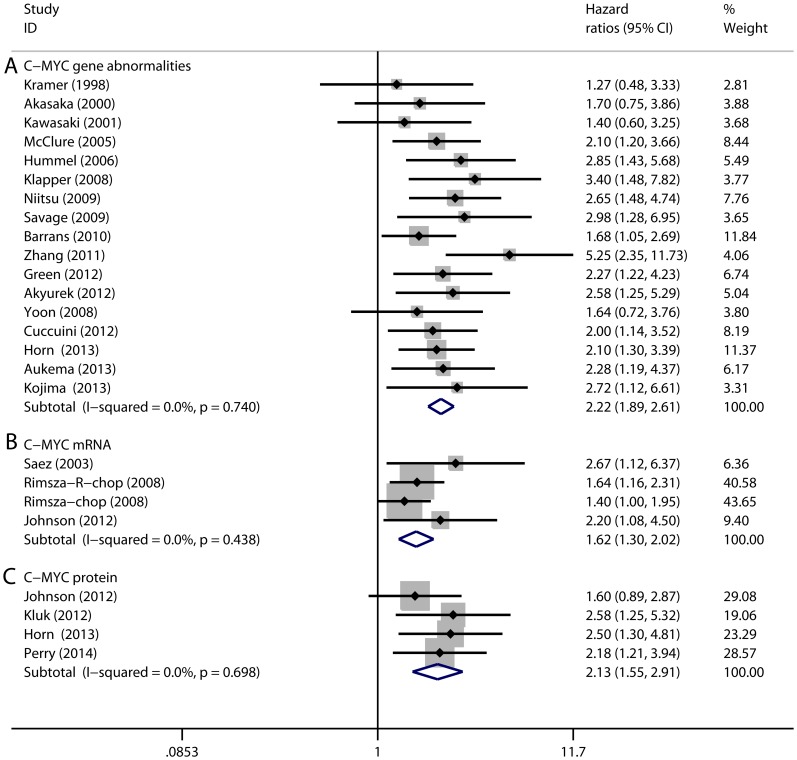
Forest plots of hazard ratios (HRs) and 95% confidence intervals (95% CIs) for overall survival endpoints in DLBCL patients with C-MYC gene abnormalities (A), overexpression of C-MYC mRNA (B) and C-MYC protein (C). Squares represent the HR of each study, and the area of each square was proportional to the weight of each study in the meta-analysis; Horizontal lines, 95% CIs; Closed diamond, pooled HRs with their 95% CIs.

**Figure 3:**
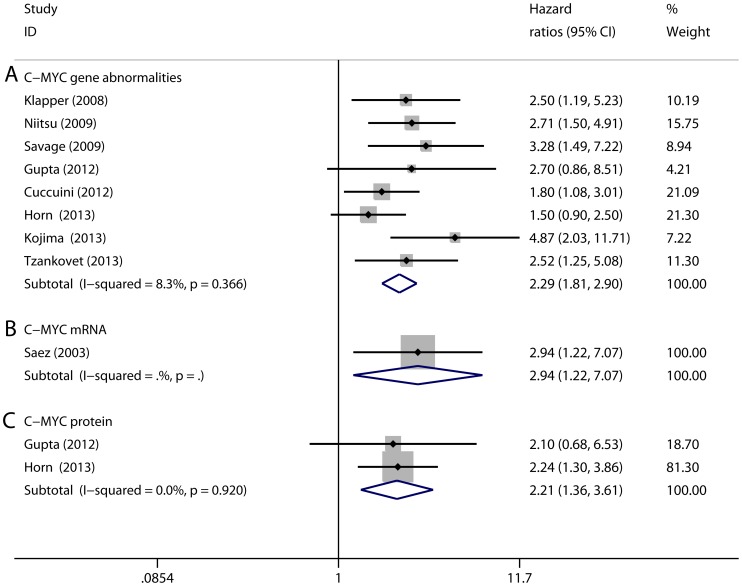
Forest plots of hazard ratios (HRs) and 95% confidence intervals (95% CIs) for event-free survival endpoints in DLBCL patients with C-MYC gene abnormalities (A), overexpression of C-MYC mRNA (B) and C-MYC protein (C). Squares represent the HR of each study, and the area of each square was proportional to the weight of each study in the meta-analysis; Horizontal lines, 95% CIs; Closed diamond, pooled HRs with their 95% CIs.

The forest plots of HRs and 95%CIs for OS or EFS endpoints in DLBCL patients are shown in Figure 2 and Figure 3. In particular, subgroup analyses were performed according to the subtypes of C-MYC aberrations (gene, protein, and mRNA) and treatment.

Initially, the prognostic value of C-MYC gene abnormalities was analyzed. Nineteen selected studies had investigated C-MYC gene abnormalities [7], [8], [9], [10], [11], [12], [13], [15], [16], [17], [20], [21], [22], [23], [25], [26], [28], [29], [30]. Among them, seventeen studies focused on only C-MYC gene translocation, whereas the other two studies examined both C-MYC gene amplification and translocation [9], [30]. For C-MYC gene abnormalities, the overall HRs for OS were 2.22 (95% CI, 1.89 to 2.61) and for EFS were 2.29 (95% CI, 1.81 to 2.90) compared with the patients without C-MYC gene abnormalities. Among the studies that investigated the prognostic value of C-MYC gene translocation, five studies had further explored whether the addition of rituximab to CHOP could reduce the prognostic value of C-MYC [7], [8], [10], [15], [20]. Our study revealed that the overall HR for OS in the R-CHOP subgroup was 2.17 (95% CI, 1.62 to 2.91), similar with the overall HR for OS in the patients who were not treated with rituximab-containing regimen (detailed results were shown in Table 3), indicating that the inferior OS conferred by C-MYC gene translocation could not be overcome by rituximab.

**Table 3:** Subgroup analyses for the prognostic values of C-MYC aberrations in DLBCL patients.

Endpoints	Studies	Pooled HRs	95% CI	*I^2^* value	Publication bias
**C-MYC gene abnormalities**					
OS	17	2.22	[1.89, 2.61]	0%	*PBegg* = 0.56; *PEgger* = 0.45
OS (translocation)	15	2.27	[1.91, 2.70]	0%	*PBegg* = 0.43; *PEgger* = 0.38
OS (translocation and amplification)	2	1.88	[1.18, 3.00]	0%	*PBegg* = 0.32; *PEgger* = 1.00
OS (R-CHOP)	5	2.17	[1.62, 2.91]	0%	*PBegg* = 0.09; *PEgger* = 0.06
OS (without R)	5	2.09	[1.48, 2.95]	2.2%	*PBegg* = 0.14; *PEgger* = 0.29
OS (adjusted)	9	2.31	[1.87, 2.86]	4.3%	*PBegg* = 0.25; *PEgger* = 0.08
EFS	8	2.29	[1.81, 2.90]	8.3%	*PBegg* = 0.07; *PEgger* = 0.06
EFS (translocation)	7	2.44	[1.87, 3.18]	8.7%	*PBegg* = 0.23; *PEgger* = 0.08
EFS (translocation and amplification)	1	1.80	[1.08, 3.01]	NA	NA
EFS (R-CHOP)	4	3.18	[2.09, 4.84]	0%	*PBegg* = 0.63; *PEgger* = 0.77
EFS (without R)	2	2.63	[1.65, 4.17]	0%	*PBegg* = 0.32; *PEgger* = 1.00
**C-MYC mRNA overexpression**					
OS	3	1.62	[1.30, 2.02]	0%	*PBegg* = 0.09; *PEgger* = 0.07
OS (R-CHOP)	2	1.73	[1.27, 2.36]	0%	*PBegg* = 0.32; *PEgger* = 1.00
EFS	1	2.94	[1.22, 7.07]	NA	NA
**C-MYC protein overexpression**					
OS	4	2.13	[1.55, 2.91]	0%	*PBegg* = 0.09; *PEgger* = 0.24
OS (R-CHOP)	2	1.93	[1.23, 3.05]	2.2%	*PBegg* = 0.32; *PEgger* = 1.00
EFS	2	2.21	[1.36, 3.61]	0%	*PBegg* = 0.32; *PEgger* = 1.00

Abbreviations: HR, hazard ratio; OS, overall survival; EFS, event-free survival; CI, confidence interval; R-CHOP, rituximab, cyclophosphamide, doxorubicin, vincristine, and prednisone; without R, treatment without rituximab; NA, not available.

Among the eligible studies, nine studies [8], [9], [13], [15], [17], [20], [21], [26], [30] that comprised 1774 patients altogether evaluated the prognostic effects of C-MYC among multivariate risk factors, such as age, IPI, and LDH. They used multivariable regression analysis to determine the independent prognostic value of C-MYC gene abnormalities. The conclusion of an ‘independent effect’ was based on the multivariate analysis. If the coefficient of a factor in a regression model did not change substantially after including other factors, it was defined as an independent factor. For this subgroup that used multivariable analysis, the pooled HR for OS was 2.31 (95% CI, 1.87 to 2.86) compared with that for the C-MYC negative patients.

Next, the prognostic value of C-MYC overexpression was analyzed. Five studies had analyzed the prognostic value of C-MYC protein overexpression [11], [14], [24], [26], [27], and three studies had investigated that of overexpression of C-MYC mRNA [18], [19], [27]. For C-MYC protein overexpression, the pooled HR for OS was 2.13 (95% CI, 1.55 to 2.91) and that for EFS was 2.21 (95% CI, 1.36 to 3.61). For C-MYC mRNA overexpression, the pooled HR for OS was 1.62 with a 95% CI from 1.30 to 2.02. Meta-analysis for EFS endpoints of C-MYC mRNA could not be performed because only one study was available. In the R-CHOP subgroup, C-MYC overexpression remained to a poor prognostic factor because the pooled HR for OS was 1.93 for protein overexpression [14], [27] and 1.73 for mRNA overexpression [18], [27].

Detailed results of subgroup analyses for C-MYC aberrations are listed in Table 3. An additional meta-regression analysis also confirmed the worse survival of C-MYC was not influenced by the difference in follow-up time (*P* > 0.05). Taken together, our study demonstrated that there was no significant heterogeneity for either EFS or OS (*I^2^* value < 50%). Publication bias tests showed no bias (*P* > 0.05). Exclusion of any single study did not alter the overall findings in the sensitivity test.

### Systematic review of the prognostic role of C-MYC amplification and the isolated C-MYC aberrations (single-hit lymphoma) in DLBCL

Apart from chromosome translocation, gene amplification was another mechanism for C-MYC overexpression that had not been widely investigated. With respect to the role of C-MYC amplification, only a few studies with diverse designs were performed. Therefore, a qualitative systematic review, rather than a quantitative meta-analysis, was performed. Only three studies with a small sample size singled out the prognosis value of C-MYC amplification in DLBCL. In the two studies that evaluated the copy-number changes by FISH, C-MYC amplification appeared to be associated with a similarly poor prognosis as C-MYC translocation [9], [30]. However, in another study that detected C-MYC amplification by array-based comparative genomic hybridization, extra copies of C-MYC were associated with poor OS and progression-free survival only in the present of concomitant del(8p), but not in all DLBCL patients [40].

Similarly, to find whether presence of double or triple hits might influence the outcome, a qualitative systematic review was performed to investigate the prognostic effects of isolated C-MYC aberrations (single-hit lymphoma). Generally, two comparison methods were applied to demonstrate the role of isolated C-MYC aberrations in DLBCL. Six studies compared single-hit patients with complex-hit cases. Among them, one study showed the complex-hit group had a worse prognosis than the single-hit group [17]; however, other studies found no difference between the two subgroups [9], [12], [22], [26], [30]. Furthermore, another three studies directly compared single-hit patients with C-MYC-negative patients. Two of them found the presence of C-MYC alone retained unfavorable prognostic significance [12], [20], but another study did not [41]. Comparisons among different studies were hampered by only a few studies available, with the diverse study designs as well as the small-size cases.

## Discussion

Thus far, the present study is the first systematic review and meta-analysis about the prognostic value of C-MYC in DLBCL. The current meta-analysis integrated 4662 DLBCL cases and strongly confirmed the role of C-MYC as a prognostic factor in DLBCL. To increase accuracy, studies were divided into several subgroups according to the nature of C-MYC aberrations and treatment. Based on this meta-analysis, several critical issues had been addressed. First, DLBCL patients with C-MYC were proven to be associated with several adverse clinical features. Second, the addition of rituximab did not seem to overcome the inferior outcome conferred by C-MYC. Moreover, after pooling the HRs adjusted by multivariate Cox models from nine studies, C-MYC appeared to maintain its independent prognostic value, regardless of other well-established factors. In light of meta-regression and subgroup analysis, the pooled results appeared not to be influenced by the length of follow-up time. In addition, the median follow-up time in all the included studies exceeded 24 months. C-MYC aberrations represented the most reproducible biomarker with unfavorable prognosis regardless of the biological test used. Screening of C-MYC should have definite prognostic meaning for DLBCL stratification, thus guaranteeing more tailored therapy.

Some questions remain uncertain regarding the prognostic role of C-MYC aberrations in DLBCL. First, a qualitative systematic review was performed on the role of C-MYC amplification due to the rarity and heterogeneity of the selected studies. It had been postulated that overexpression of C-MYC by amplification had a similar effect to up-regulation by C-MYC translocation in DLBCL [42], [43], [44]. However, different studies with small-size cases appeared to be inconsistent. Similarly, the prognostic effects of isolated C-MYC aberrations (single-hit lymphoma) also remained controversial due to the limited studies with inconsistent conclusion. In the future, prospective studies with large sample size or a patient-level meta-analysis need to be conducted to address these issues.

Second, assessment of C-MYC aberrations in pathology specimens is becoming increasingly important in the routine clinical practice. Statistical analysis has demonstrated there is a significant correlation between deregulation of C-MYC protein and mRNA with C-MYC gene abnormalities [43], [44], [45]. Unfortunately, in contrast to the ease in detecting C-MYC gene translocation, it is not so straightforward to define DLBCL patients with C-MYC mRNA or protein overexpression because markedly different cut-off values and methods were apparent between centers. Some studies established cut-off values (50% or 70%) for classifying tumors as the lowest C-MYC IHC score that captured all cases with a confirmed C-MYC translocation [14], [45], while others used a lower threshold (40%) which was set based on the relationship between C-MYC protein and survival, not the presence of a translocation [27]. The difference in tissue processing, inter-observer variability and cut-off values contributes to poor reproducible results of the mRNA/protein expression levels among different institutions. Therefore, at this point, it is difficult to make definitive recommendations regarding the optimal cut-off points for C-MYC for general use, as these needs to be validated in large prospective cohorts of DLBCL patients. Nevertheless, the conclusion of this meta-analysis is still valid. Standardization and validation of current assays and agreement upon the techniques to quantitate C-MYC mRNA or protein are at urgent need for its widespread application in clinical practice.

In conclusion, this systematic review and meta-analysis underscores the prognostic value of C-MYC in DLBCL. Screening of C-MYC aberrations could enable the early identification of DLBCL patients with poor prognosis and guide a more tailored therapy. Further well-designed clinical trials should be warranted to address the uncertain problems due to the current limited studies.

## Supporting Information

Checklist S1 **PRISMA 2009 Checklist.**
(DOC)
